# Simultaneous Estimation of Metformin and Pioglitazone by Ultraviolet Spectrophotometry

**DOI:** 10.4103/0250-474X.73940

**Published:** 2010

**Authors:** Laxmi Goswami, S. Mukhopadhyay, S. Durgapal

**Affiliations:** Department of Pharmacy, G. R. D. Institute of Management and Technology, 214-Rajpur Road, Dehradun-248 009, India; 1 Department of Pharmacy, Abhilashi College of Pharmacy, Mandi-175 008, India

**Keywords:** Derivative spectrophotometery, metformin, pioglitazone, q-analysis, simultaneous equation method

## Abstract

This work deals with the simultaneous estimation of metformin hydrochloride and pioglitazone hydrochloride in a bilayered tablet dosage form, without prior separation by two techniques. The methods employed are derivative spectrophotometery and Q analysis. The absorption maxima at 231 nm and 269 nm were used for the estimation of metformin and pioglitazone, respectively. Both the drugs and their mixture obey Beer-Lamberts law at selected wavelength at given concentration range. The result of analysis has been validated statistically and recovery studies confirmed the accuracy of the proposed method. The proposed procedures are simple, rapid, require no separation steps and can be used for the routine analysis of both drugs.

Combined tablets of metformin and pioglitazone hydrochloride are available containing 500 mg of metformin and 15 mg of pioglitazone hydrochloride. Because metformin’s insulin-sensitizing effect occurs mainly at the liver, combination with thiazolidinediones (TZDs), which mainly sensitize muscle to insulin-mediated glucose uptake, is a rational therapeutic strategy[1]

The aim of this paper was to explore the possibility of using techniques of absorbance ratio or Q-analysis method and derivative spectrophotometery methods for quantifying metformin and pioglitazone simultaneously in their mixture form. The advantage of these proposed methods is that no separation is required. The proposed procedures are simple, rapid and act as convenient alternative to HPLC method[2]

The absorption spectra of the reference and test solutions were recorded over the range of 200-400 nm keeping the solutions in 1 cm quartz cells using Shimadzu UV/Vis double beam spectrophotometer model 1700 Pharmaspec.

Gift samples of pure metformin and pioglitazone hydrochloride were procured from Zydus Cadila, Sikkim. Combined metformin and pioglitazone tablets (Pioz*MF-15 containing metformin-500 mg and pioglitazone-15 mg and manufactured by USV Limited) were purchased from a local pharmacy. Analytical reagent grade hydrochloric acid, N,N-dimethyl formamide, distilled water was used as solvent. Stock solutions (500 μg/ml) of metformin and pioglitazone hydrochloride were prepared by dissolving separately 50 mg in 10 ml of N,N-dimethyl formamide in 100 ml volumetric flasks, and the volume was made up to 100 ml with 0.1 N hydrochloric acid.

In the quantitative assay of two components by Q-analysis method[3], absorbance were measured at two wavelengths, one being the isoabsorptive point and other being the wavelength of maximum absorption of one of the two components. From the overlain spectra of metformin and pioglitazone, 247.5 nm was the isoabsorptive point for both the drugs and other at 231 nm, the λ_max_for metformin was selected (fig. 1). The method employs Q-values, concentrations of drugs in sample solutions were determined using equation C_1_= (Q_0_-Q_2_)/(Q_1_-Q_2_)×(A/a_1_) for pioglitazone and C_2_= (Q_0_-Q_2_)/(Q_2_-Q_1_)×(A/a_2_) for metformin, where Q_0_= absorption of sample at 231 nm/absorption of sample at 247.5 nm, Q_1_= absorptivity of pioglitazone at 231 nm/absorptivity of pioglitazone at 247.5 nm, Q_2_= absorptivity of metformin at 231 nm/absorptivity of metformin at 247.5 nm, A= absorption of sample at isoabsorptive point and a_1_and a_2_= absorptivities of pioglitazone and metformin respectively at isoabsorptive point.

**Fig. 1:**
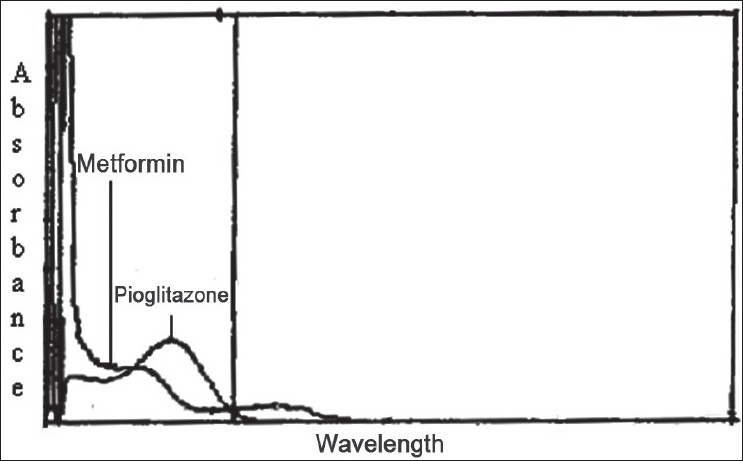
Overlain Spectra of metformin and pioglitazone

In the derivative spectrophotometery[4] method solutions of 10 μg/ml of metformin and pioglitazone were prepared separately. Both the solutions were scanned in the spectrum mode from 400 to 200 nm. The absorption spectra thus obtained were derivatized from first to fourth order. First order derivative was selected for analysis of both the drugs. The zero crossing wavelengths, 269 and 231 nm were selected for pioglitazone and metformin respectively (fig. 2).

**Fig. 2:**
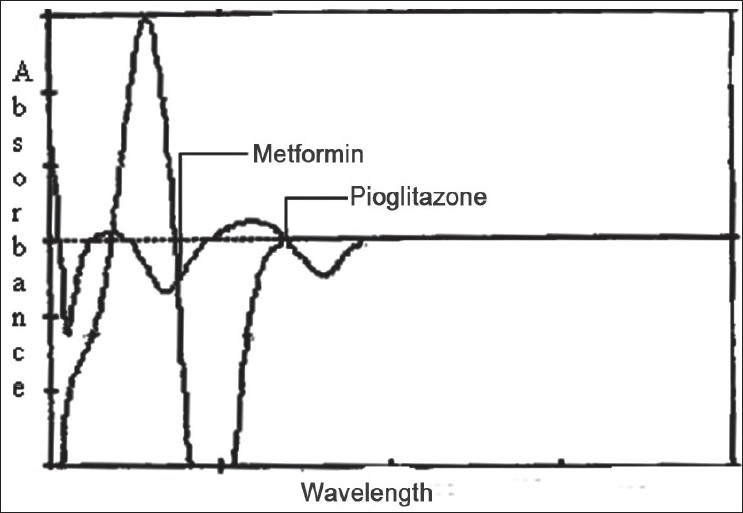
Derivative spectrophotometry of metformin and pioglitazone

For preparation of calibration curves five mixed standards having concentrations 0.5, 1, 1.5, 3 and 4.5 μg/ml of pioglitazone and 16.5, 33, 50, 100 and 150 μg/ml of metformin, respectively were prepared and scanned in the spectrum mode from 400 to 200 nm. The absorption spectra so obtained were derivatized to obtain first derivative order spectra. The absorbance of pioglitazone and metformin were measured at 269 and 231 nm, respectively and working calibration curves of both the drugs were plotted separately. The concentration of individual drug present in the mixture was determined against calibration curve in quantitation mode.

Twenty tablets were weighed accurately. The average weight was determined and then ground to a fine powder. A quantity equivalent to 15 mg of pioglitazone and 500 mg of metformin were transferred to a 100 ml volumetric flask. The contents were ultrasonicated for 10 min with solvent, made to volume and filtered through Whatmann filter paper. The solution was further diluted with solvent, to give concentration of μg/ml of pioglitazone and metformin, respectively. Absorbance of these solutions was measured at 269 nm and 231 nm, and concentration of pioglitazone and metformin obtained from standard calibration curve. Results of tablet analysis of the tablet formulation are reported in Table 1. Accuracy, reproducibility and precision of the proposed method were studied with the help of recovery studies that was carried out by addition of standard drug solution to pre-analyzed sample.

**TABLE 1:** ANALYSIS OF TABLET FORMULATION

Method	Label Claim (mg/tablet)		Percent found (±SD)	
	Pio	Met	Pio	Met
DS	15	500	99.13±0.978	99.90±0.637
QA	15	500	99.53±1.084	99.92±0.714

DS denotes derivative spectrophotometery and QA denotes Q-analysis. Pio represents pioglitazone hydrochloride and Met represents metformin hydrochloride. SD denotes standard deviation.

The proposed methods for simultaneous estimation of pioglitazone and metformin dosage forms were found to be simple, accurate, economical and rapid. In both the methods, the values of coefficient of variation were satisfactorily low and recovery was close to 100% (Table 2)for both the drugs. Hence it can be employed for routine analysis in quality control laboratories.

**TABLE 2:** RECOVERY STUDY DATA OF TABLET FORMULATION

Method	Level of % recovery	% Recovery found		Standard deviation		Standard error	
DS QA		Pio	Met	Pio	Met	Pio	Met
	80	99.87	100.27	0.554	1.286	0.320	0.743
	100	99.85	99.20	0.528	1.311	0.305	0.757
	120	99.91	99.47	0.581	1.249	0.337	0.721
	80	99.71	99.33	0.140	1.331	0.081	0.768
	100	99.85	99.20	0.466	1.587	0.275	0.916
	120	99.91	99.47	0.689	1.514	0.408	0.874

DS denotes derivative spectrophotometery and QA denotes Q-analysis. Pio represents pioglitazone hydrochloride and Met represents metformin hydrochloride.
